# By Belgians for Belgians

**Published:** 1915-02-13

**Authors:** M. A. Childers

**Affiliations:** Hon. Secretary.


### THE EDITOR'S LETTER-BOX.

### "By Belgians for Belgians."

*To the Editor of* The Hospital.

Sir,—During this terrible winter, in England and France all women are busy making clothes to send to their men at the Front. But the Belgian soldiers are fighting among the ruins of their old homes, and there is no one to send them warmth and comfort. The Belgian women —now refugees in England—would gladly work too for their soldiers, but they have nothing left to give but their labour, and no means to buy material. The Chelsea War Refugees' Committee can find Belgian ladies to make up wool and flannel into warm garments for the Belgian Army. They would be grateful for donations, however small, either of money or of grey and natural flannel, and of wool. The latter may be of any neutral or dark colour. Remnants of other materials suitable for women's and children's clothes will also be most welcome. All gifts should be sent to Mrs. Ernest Davies, c/o Chelsea War Refugees' Committee, Crosby Hall, Chelsea, and will be promptly acknowledged if the sender will kindly enclose name and address. The garments will be quickly made up and sent off without delay. The work will be done by Belgians for Belgians, and will give happiness to many a refugee.—Yours truly,

M. A. Childers, Hon. Secretary.

